# Iron absorption and phosphate-lowering effects of ferric citrate hydrate are not influenced by gastric acid secretion inhibitors in patients with chronic kidney disease: a retrospective post hoc analysis

**DOI:** 10.1007/s11255-022-03287-1

**Published:** 2022-07-11

**Authors:** Kyoko Ito, Keitaro Yokoyama

**Affiliations:** 1 Medical Affairs Department, Torii Pharmaceutical Co., Ltd., 3-4-1 Nihonbashi-Honcho, Chuo-ku, Tokyo, 103-8439 Japan; 2 Department of Health Science, The Graduate School, The Jikei University School of Medicine, 3-25-8 Nishi-Shinbashi, Minato-ku, Tokyo, 105-8471 Japan

**Keywords:** Ferric citrate hydrate, Iron deficiency anemia, Chronic kidney disease, Gastric acid secretion inhibitors, Proton pump inhibitor, Histamine-2 receptor antagonist

## Abstract

**Background:**

Ferric citrate hydrate (FC), an oral iron product is approved as iron preparation for iron deficiency anemia and phosphate binder for chronic kidney disease (CKD). We investigated whether gastric acid secretion inhibitors (GASI) influenced on iron absorption and phosphate-lowering effects of FC.

**Methods:**

Two phase 3 studies of FC for treatment of hyperphosphatemia in CKD patients (non-dialysis-dependent, 12 weeks, and hemodialysis, 52 weeks), were retrospectively analyzed. Patients were divided into with or without concomitant GASI and levels of iron- and phosphate-related parameters were analyzed.

**Results:**

In non-dialysis study (FC, 60 patients; placebo, 30 patients), 14 FC patients and 14 placebo patients used GASI. No significant differences were found between the FC and placebo groups for adjusted mean differences (95% CI) of changes from baseline to end of treatment (EOT) in serum ferritin [104.84 ng/mL (35.97, 173.71) with GASI vs 145.30 ng/mL (96.34, 194.25) without GASI, *P* = 0.34], and transferrin saturation (TSAT) [12.56% (− 0.83, 25.95) with GASI vs 18.56% (8.15, 28.98) without GASI, *P* = 0.49]. In hemodialysis study, 95/180 patients used GASI. Mean changes (SD) from baseline to EOT in serum ferritin were 166.32 ng/mL (153.70) with GASI and 155.16 ng/mL (139.47) without GASI, and for TSAT were 16.60% (19.44) with GASI and 16.02% (18.81) without GASI. In both studies, there were no differences in the changes from baseline to EOT in serum phosphate between with and without GASI cohorts.

**Conclusion:**

GASI did not influence on the changes in serum ferritin, TSAT and serum phosphate by FC administration.

## Introduction

Iron is important for various biological processes and, therefore, its homeostasis is tightly regulated. Ingested iron is dissolved at a low pH by gastric acid secreted in the stomach, which is an important process for its effective absorption in the small intestine [1]. Patients taking gastric acid secretion inhibitors (GASI), such as proton pump inhibitors and histamine-2 receptor antagonists, showed decreased iron absorption and increased risk of iron deficiency, which were dose- and treatment duration-dependent [2]. The incidence rate of iron deficiency was high in patients who underwent gastrectomy or gastric bypass surgery [3, 4]. These findings demonstrate that the low pH of gastric acid is essential for dietary iron absorption.

Ferric citrate hydrate (FC, Riona^®^, Torii Pharmaceutical Co., Ltd., Tokyo, Japan) is an oral iron-based phosphate binder, and the ferric iron of FC binds to dietary phosphorus to form an insoluble complex that promotes the fecal excretion of phosphorus [5], thereby effectively lowering serum phosphate in patients with chronic kidney disease (CKD) who are non-dialysis-dependent [6] or undergoing dialysis [7–9]. In addition, ferric iron from FC was enzymatically reduced to ferrous iron and absorbed in the small intestine [10, 11], which improved anemia in patients with iron deficiency anemia [12]. Therefore, FC is also indicated for use as an iron preparation for patients with iron deficiency anemia in Japan. In the USA, ferric citrate (Auryxia^®^; Akebia Therapeutics Inc., Cambridge, MA, USA) is indicated for use as a phosphate binder in patients with CKD undergoing dialysis and as an iron preparation to treat iron deficiency anemia in patients with CKD not undergoing dialysis. In Taiwan, ferric citrate (Nephoxil^®^, Panion & BF Biotech Inc., Taipei, Taiwan) is indicated for use as a phosphate binder in patients with CKD undergoing hemodialysis.

FC needs to be dissolved in the stomach to exert its phosphate-lowering and iron absorption effects. In a previous study, FC showed a similar phosphate-lowering effect in CKD patients with hyperphosphatemia undergoing hemodialysis when treated with or without a concomitant histamine-2 receptor antagonist [13]. These results suggest FC can be dissolved, even when gastric acid secretion has been inhibited, and has a consistent phosphate-lowering effect regardless of the pH level of the stomach.

The suppression of gastric acid by omeprazole (proton pump inhibitor) was reported to impair the absorption of an oral ferrous iron preparation (ferrous sulfate) in a rat model and in patients with iron deficiency anemia [14, 15]; however, no study has investigated the absorption of oral ferric iron preparations. Therefore, it is unclear whether iron absorption from FC is affected by GASI use.

In this study, we retrospectively investigated data from two phase 3 clinical studies to determine the influence of GASI on the iron absorption and phosphate-lowering effects of FC in CKD patients with hyperphosphatemia who were non-dialysis-dependent [6] or who were undergoing hemodialysis [7].

## Materials and methods

### Study design

This was a retrospective study using data from two previous clinical studies to investigate FC in CKD patients with hyperphosphatemia. The GBA4-4 study was a 12-week, phase 3, multicenter, randomized, double-blind, placebo-controlled, dose-titration study in non-dialysis-dependent CKD patients [6], and the GBA4-6 study was a 52 week, phase 3, multicenter, open-label, dose-titration study in CKD patients undergoing hemodialysis [7].

### Patients

Detailed inclusion and exclusion criteria and interventions for these studies were described previously [6, 7]. Briefly, CKD patients with hyperphosphatemia, who were ≥ 20 years old when informed consent was provided, were recruited. Patients included in GBA4-4 were at CKD stages 3 − 5 and received standard conservation therapy and had a serum phosphate level ≥ 5.0 mg/dL and < 8.0 mg/dL at 2 weeks before, 1 week before, or at FC treatment initiation. Patients included in GBA4-6 were undergoing hemodialysis three times a week for ≥ 3 months before treatment initiation and had a serum phosphate level ≥ 3.5 mg/dL and < 10.0 mg/dL with hyperphosphatemia therapy or ≥ 6.1 mg/dL and < 10.0 mg/dL without hyperphosphatemia therapy at FC treatment initiation. Patients who had gastrointestinal disease and previous gastrectomy or duodenectomy were excluded. FC (Riona^®^ 250 mg, containing approximately 60 mg of ferric iron) was taken orally three times a day immediately after meals. The starting dose was 1500 mg/day and the dose was adjusted up to 6000 mg/day to achieve a serum phosphate level ≥ 2.5 mg/dL and ≤ 4.5 mg/dL (GBA4-4) or ≥ 3.5 mg/dL and ≤ 6.0 mg/dL (GBA4-6). The FC treatment periods were 12 weeks (GBA4-4) and 52 weeks (GBA4-6).

The use of GASI [proton pump inhibitors (Omeprazole, Lansoprazole, and Rabeprazole Sodium) or histamine-2 receptor antagonists (Cimetidine, Ranitidine Hydrochloride, Famotidine, Nizatidine, Roxatidine Acetate Hydrochloride, and Lafutidine)] from 4 weeks before treatment initiation until the end of treatment (EOT, end of the study or treatment discontinuation) was determined for all included patients. Patients were divided into two cohorts: patients treated with or without GASI. For the GBA4-4 study, patients were divided into two cohorts (with or without GASI) for each treatment group (FC and placebo groups).

### Analysis of iron-related and mineral and bone disorder-related parameters

From each study, iron-related parameters, including the levels of serum iron, serum ferritin, total iron-binding capacity (TIBC), transferrin saturation (TSAT), and hemoglobin (Hb), were collected from the safety analysis set, and mineral and bone disorder (MBD)-related parameters, including serum phosphate, serum corrected calcium (cCa), intact parathyroid hormone (iPTH), and calcium–phosphate calculated variable (Ca*P), were collected from the efficacy analysis set (see “Statistics”). These evaluation items were measured at screening, baseline, and at pre-determined intervals throughout the study period.

### Statistics

Iron-related parameters and Hb were analyzed from data of patients who received FC at least once and data for safety evaluation items were collected (safety analysis set). MBD-related parameters were analyzed from the data of patients who received FC and data for efficacy evaluation items were collected at least once (efficacy analysis set). In each study, changes from baseline to the EOT (at 12 weeks for GBA4-4, 52 weeks for GBA4-6, or at discontinuation) were calculated for all safety and efficacy evaluation items. For the GBA4-4 study, adjusted mean differences [(least square mean of FC) − (least square mean of placebo)] and 95% confidence intervals (CI) were calculated for differences between FC and placebo groups in changes from baseline to EOT and compared using analysis of covariance. For the GBA4-6 study, changes from baseline were summarized descriptively. SAS ver. 9.4 (SAS Institute Inc., Cary, NC, USA) was used for all statistical analyses.

## Results

### Use of GASI

**Fig. 1:**
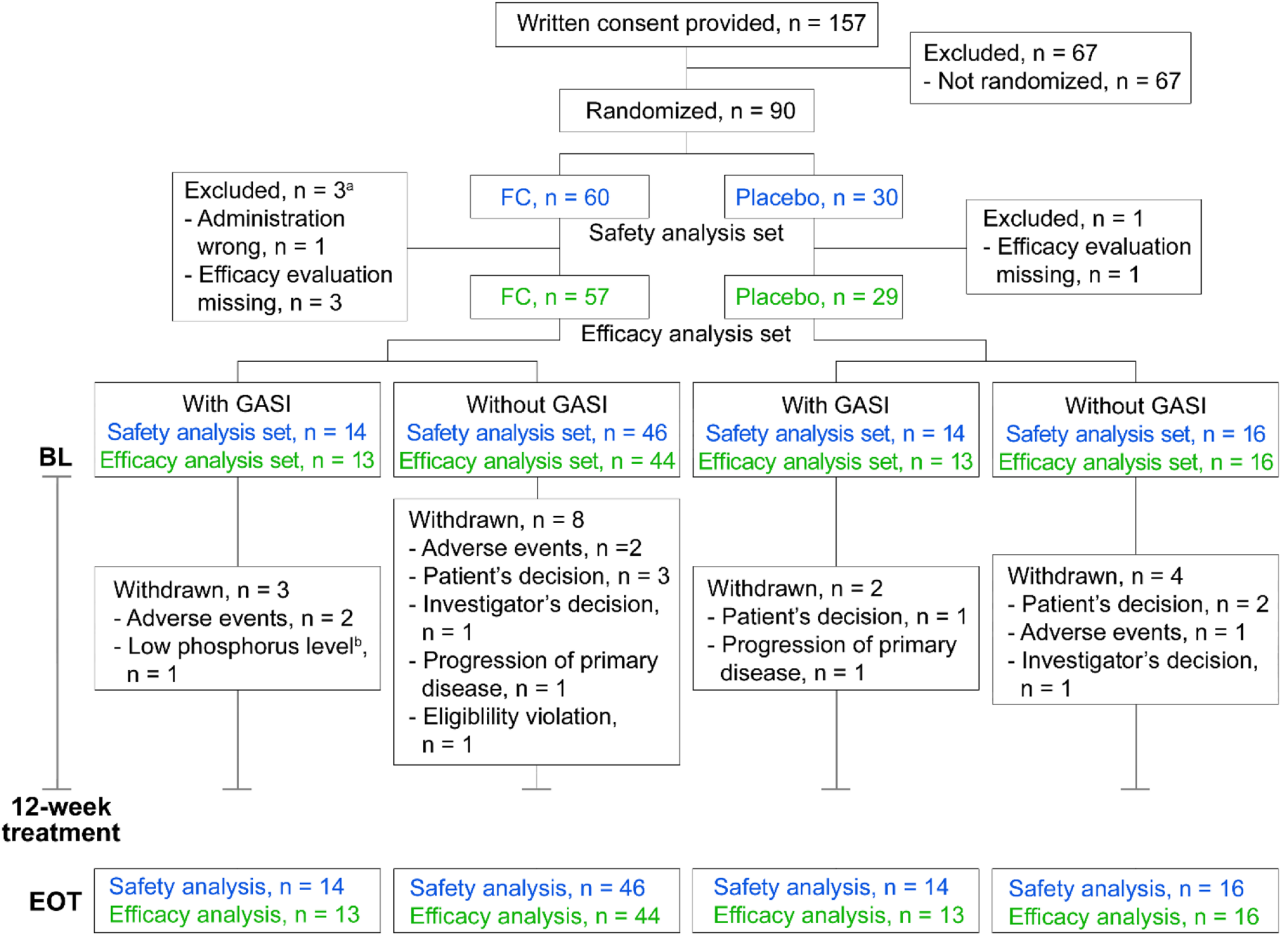
Patient flow in the GBA4-4 study. Flow of non-dialysis-dependent patients from the GBA4-4 study. **a** A patient may have multiple reasons to withdraw. **b** Serum phosphate level was < 2.5 mg/dL in two consecutive investigations after FC treatment initiation. *BL* baseline, *EOT* end of treatment, *FC* ferric citrate hydrate, *GASI* gastric acid secretion inhibitor

**Fig. 2:**
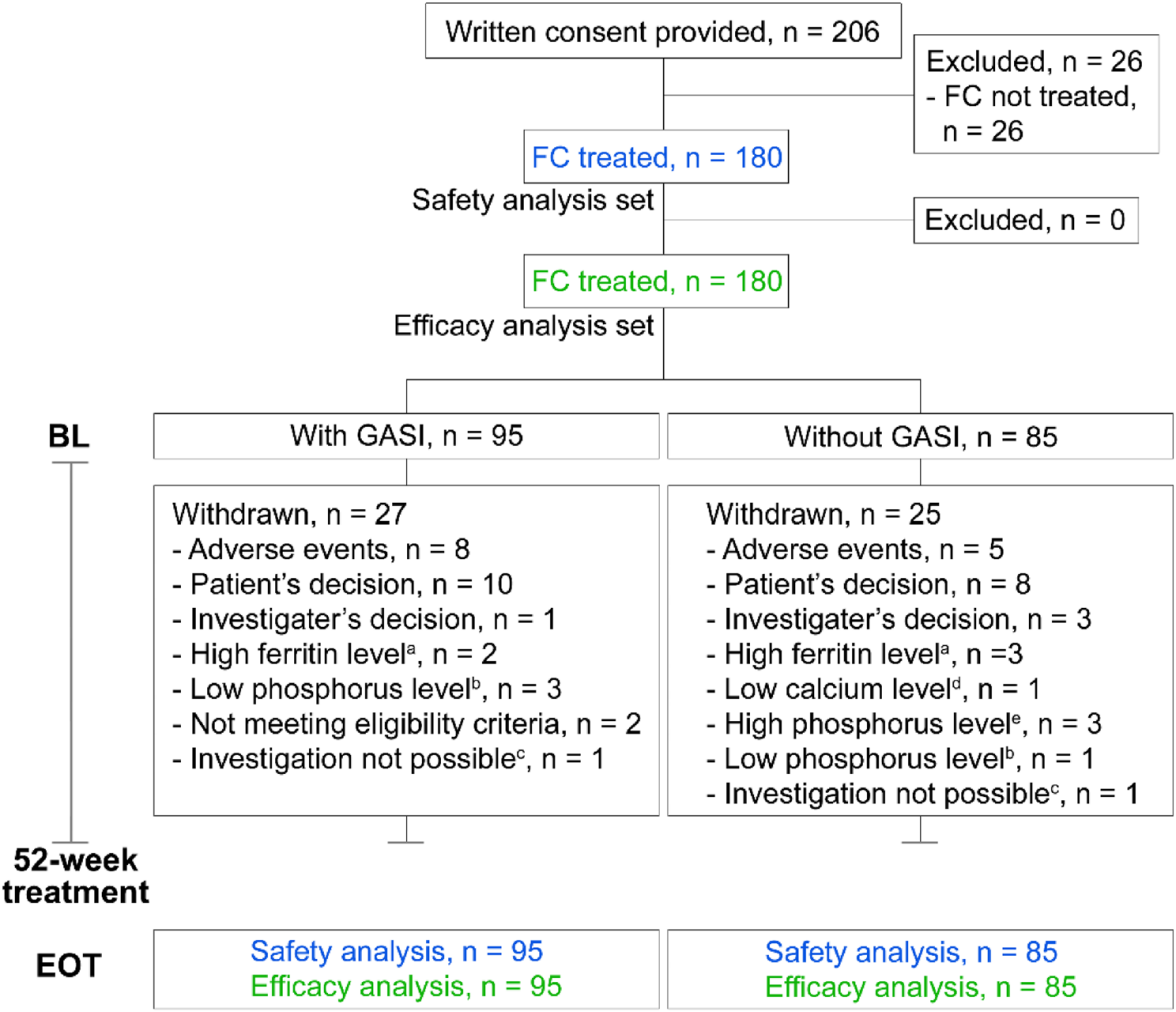
Patient flow in the GBA4-6 study. Flow of patients undergoing hemodialysis from the GBA4-6 study. **a** Ferritin level ≥ 800 ng/mL. **b** Serum phosphate level was < 3.0 mg/dL in two consecutive investigations. **c** Investigation was not possible for patient’s reason. **d** Serum corrected calcium level was < 7.5 mg/dL in two consecutive investigations. **e** Serum phosphate level was ≥ 10.0 mg/dL in two consecutive investigations. *BL* baseline, *EOT* end of treatment, *FC* ferric citrate hydrate, *GASI* gastric acid secretion inhibitor

Data were collected from 90 non-dialysis-dependent CKD patients from GBA4-4. All patients were randomized 2:1 to FC (*n* = 60) or placebo (*n* = 30) groups and were included in the safety analysis. Patients, whose administration was wrong or effective evaluation was missing, were excluded from the efficacy analysis (*n* = 3 from the FC group and *n* = 1 from the placebo group). Approximately 25% of patients in the FC group (13/60 patients) and 50% in the placebo group (13/30 patients) were treated with GASI (Fig. 1). Data were collected from 180 patients in GBA4-6 undergoing hemodialysis. All of these patients were treated with FC and all efficacy evaluation items were present; therefore, all data were analyzed for safety and efficacy. Approximately half of these (95/180 patients) were treated with GASI (Fig. 2).

### Influence of GASI on non-dialysis-dependent patients (GBA4-4)

**Fig. 3:**
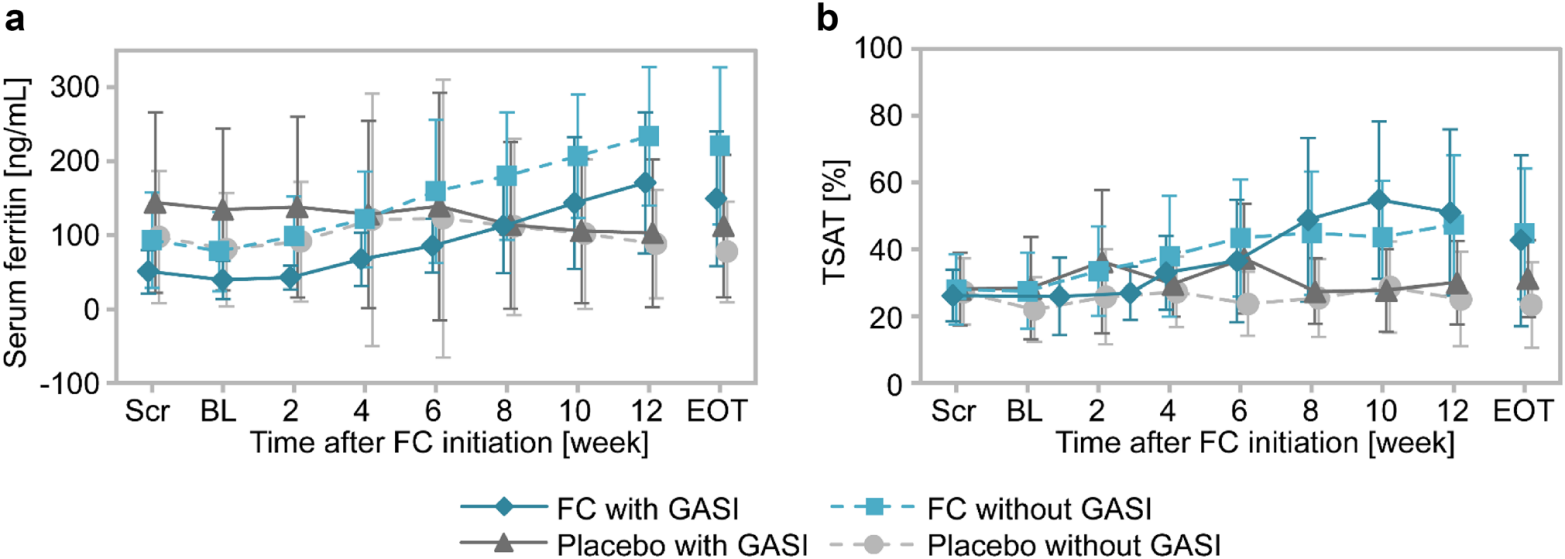
Time-course changes in serum ferritin and TSAT in non-dialysis-dependent patients from the GBA4-4 study (safety analysis set). Time-course changes in serum ferritin (**a**) and TSAT (**b**). Blue lines, FC group; gray lines, placebo group; solid lines, with GASI cohort; broken lines, without GASI cohorts. Data are the mean ± standard deviation. *BL* baseline, *EOT* end of treatment, *FC* ferric citrate hydrate, *GASI* gastric acid secretion inhibitor, *Scr* screening, *TSAT* transferrin saturation

Time-course changes in serum ferritin and TSAT levels are shown in Fig. 3. The levels of serum ferritin gradually increased in the FC group after FC treatment initiation and this increase was also observed in with and without GASI cohorts (Fig. 3a). TSAT levels were also increased with time in the FC group, and the increase was similar in with and without GASI cohorts (Fig. 3b). The changes from baseline to EOT in these iron-related parameters and Hb are summarized in Table 1. Serum ferritin increased from baseline to EOT in the FC group regardless of GASI use and the adjusted mean differences (95% CI) compared with the placebo group were 104.84 ng/mL (35.97, 173.71) with GASI and 145.30 ng/mL (96.34, 194.25) without GASI use. No significant interaction with GASI use was detected (*P* = 0.34). The adjusted mean differences (95% CI) in changes in TSAT between the FC and placebo groups were 12.56% (− 0.83, 25.95) with GASI and 18.56% (8.15, 28.98) without GASI use. There were no significant interactions for GASI use (*P* = 0.49). Similarly, an analysis of covariance did not detect any significant influence of GASI use on serum iron, TIBC, and Hb.

**Table 1:** Changes in iron-related parameters in non-dialysis-dependent patients from the GBA4-4 study (safety analysis set)

Mean (SD)	FC, *n* = 60						Placebo, *n* = 30						FC − Placebo^a^		
	With GASI, *n* = 14			Without GASI, *n* = 46			With GASI, *n* = 14			Without GASI, *n* = 16			With GASI	Without GASI	Interaction
	BL	EOT	Change	BL	EOT	Change	BL	EOT	Change	BL	EOT	Change	FC − placebo, adjusted mean difference^b^ (95% CI)		*P* value^c^
Serum iron [µg/dL]	69.9 (30.2)	97.0 (52.0)	27.1 (66.0)	72.0 (26.6)	107.7 (45.2)	35.7 (42.6)	72.4 (41.1)	79.9 (32.1)	7.5 (35.4)	56.9 (21.0)	61.3 (31.4)	4.3 (24.8)	18.2 (− 12.4, 48.8)	39.8 (15.8, 63.8)	0.27
Serum ferritin [ng/mL]	39.41 (25.60)	149.24 (91.11)	109.83 (104.46)	78.00 (53.43)	220.68 (106.15)	142.68 (94.28)	134.75 (109.35)	112.26 (96.13)	− 22.49 (74.85)	80.81 (76.49)	77.39 (67.89)	− 3.43 (43.58)	104.84 (35.97, 173.71)	145.30 (96.34, 194.25)	0.34
TIBC [µg/dL]	273.4 (37.2)	238.6 (34.8)	− 34.7 (29.6)	267.9 (44.7)	243.0 (35.2)	− 24.8 (27.8)	254.0 (40.1)	257.0 (34.2)	3.0 (19.2)	271.6 (54.0)	270.1 (40.3)	− 1.5 (19.0)	− 31.1 (− 46.7, − 15.5)	− 24.6 (− 36.5, − 12.7)	0.51
TSAT [%]	25.94 (11.52)	42.59 (25.58)	16.64 (30.13)	27.61 (11.34)	44.67 (19.53)	17.07 (17.56)	28.37 (15.28)	31.23 (11.44)	2.86 (14.68)	22.04 (9.59)	23.35 (12.75)	1.31 (9.75)	12.56 (− 0.83, 25.95)	18.56 (8.15, 28.98)	0.49
Hb [g/dL]	10.08 (1.06)	10.80 (2.27)	0.72 (1.90)	10.30 (1.57)	10.68 (1.77)	0.38 (1.65)	10.99 (1.55)	11.02 (1.14)	0.03 (0.93)	10.11 (0.79)	10.19 (1.27)	0.08 (0.93)	0.39 (− 0.71, 1.49)	0.37 (− 0.46, 1.20)	0.98

*BL* baseline, *CI* confidence interval, *EOT* end of treatment, *FC* ferric citrate hydrate, *GASI* gastric acid secretion inhibitor, *Hb* hemoglobin, *SD* standard deviation, *TIBC* total iron-binding capacity, *TSAT* transferrin saturation

^a^Analysis of covariance (covariate, baseline); model: change from baseline = [treatment] [type] [baseline] [treatment] * [type]

^b^Adjusted mean difference = [least square mean of FC] − [least square mean of placebo]

^c^*P* value: test for [adjusted mean difference of with GASI] vs [adjusted mean difference of without GASI]

**Fig. 4:**
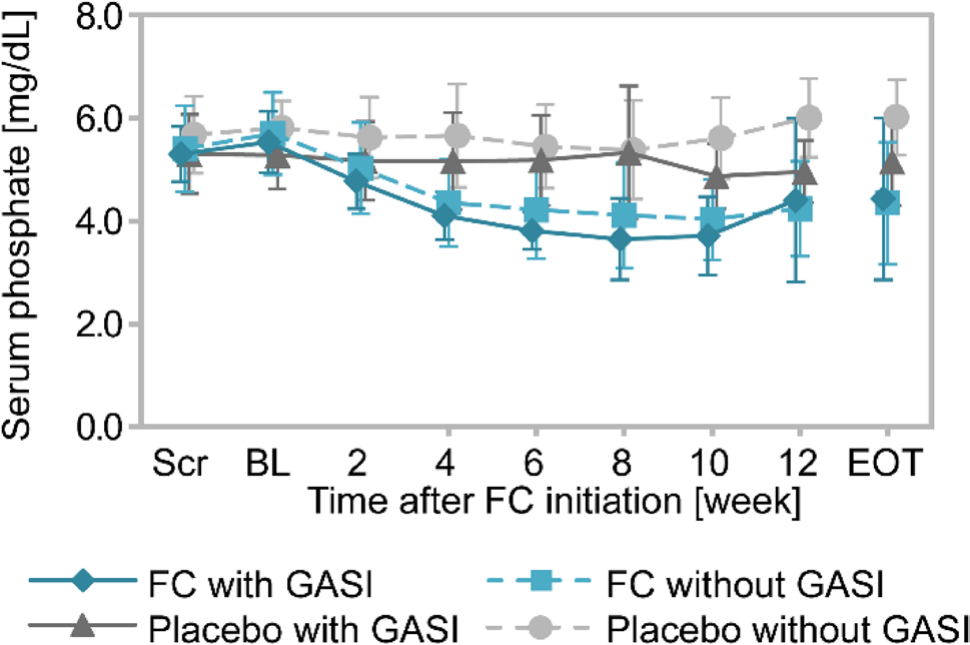
Time-course changes in serum phosphate in non-dialysis-dependent patients from the GBA4-4 study (efficacy analysis set). Time-course changes in serum phosphate. Blue lines, FC group; gray lines, placebo group; solid lines, with GASI cohort; broken lines, without GASI cohorts. Data are the mean ± standard deviation. *BL* baseline, *EOT* end of treatment, *FC* ferric citrate hydrate, *GASI* gastric acid secretion inhibitor, *Scr* screening

Time-course changes in serum phosphate are shown in Fig. 4. Serum phosphate levels were similar in the FC and placebo groups at baseline, and they were lower in the FC group compared with the placebo group when the treatment advanced. There was no notable difference in these parameters between with and without GASI cohorts. The changes in MBD-related parameters from baseline to EOT in the FC and placebo groups with and without GASI use are summarized in Table 2. The adjusted mean differences (95% CI) in the reduction of serum phosphate in the FC group compared with the placebo group were − 0.85 mg/dL (− 1.70, − 0.01) with GASI and − 1.61 mg/dL (− 2.23, − 0.98) without GASI, indicating no significant interaction with GASI use (*P* = 0.16). Similarly, there was no interaction with GASI use in Ca*P change (adjusted mean differences (95% CI) − 6.37 (mg/dL)^2^ (− 13.17, 0.43) with GASI and − 12.66 (mg/dL)^2^ (− 17.68, − 7.64) without GASI, *P* = 0.14). GASI use did not influence changes in serum cCa by FC treatment, but did influence changes in iPTH [adjusted mean differences (95% CI): 0.4 pg/mL (− 79.5, 80.3) with GASI and − 116.3 pg/mL (− 175.5, − 57.2) without GASI, *P* = 0.02].

**Table 2:** Changes in mineral and bone disorder-related parameters in non-dialysis-dependent patients from the GBA4-4 study (efficacy analysis set)

Mean (SD)	FC, *n* = 57						Placebo, *n* = 29						FC − Placebo^a^		
	With GASI, *n* = 13			Without GASI, *n* = 44			With GASI, *n* = 13			Without GASI, *n* = 16			With GASI	Without GASI	Interaction
	BL	EOT	Change	BL	EOT	Change	BL	EOT	Change	BL	EOT	Change	FC − placebo, adjusted mean difference^b^ (95% CI)		*P* value^c^
Serum P [mg/dL]	5.53 (0.59)	4.43 (1.57)	− 1.10 (1.20)	5.70 (0.80)	4.35 (1.18)	− 1.35 (1.31)	5.28 (0.64)	5.15 (0.85)	− 0.13 (0.65)	5.81 (0.52)	6.01 (0.73)	0.21 (0.67)	− 0.85 (− 1.70, − 0.01)	− 1.61 (− 2.23, − 0.98)	0.16
Serum cCa [mg/dL]	8.74 (0.64)	8.96 (0.41)	0.22 (0.54)	8.58 (0.48)	8.78 (0.61)	0.20 (0.51)	8.61 (0.45)	8.67 (0.34)	0.06 (0.31)	8.54 (0.44)	8.48 (0.48)	− 0.06 (0.40)	0.21 (− 0.13, 0.55)	0.27 (0.02, 0.53)	0.77
iPTH [pg/mL]	317.2 (478.8)	273.1 (359.5)	− 44.1 (135.1)	321.0 (260.1)	251.1 (179.6)	− 70.0 (134.4)	223.2 (129.2)	203.5 (139.0)	− 19.7 (71.2)	312.3 (202.6)	361.0 (280.7)	48.7 (125.1)	0.4 (− 79.5, 80.3)	− 116.3 (− 175.5, − 57.2)	0.02
Ca*P [(mg/dL)^2^]	48.35 (6.43)	39.66 (13.82)	− 8.69 (10.53)	48.86 (7.11)	37.86 (9.17)	− 11.0 (10.56)	45.39 (5.66)	44.51 (6.66)	− 0.88 (5.28)	49.54 (4.26)	50.87 (5.67)	1.34 (4.87)	− 6.37 (− 13.17, 0.43)	− 12.66 (− 17.68, − 7.64)	0.14

*BL* baseline, Ca*P calcium–phosphate product, *cCa* corrected calcium, *CI* confidence interval, *EOT* end of treatment, *FC* ferric citrate hydrate, *GASI* gastric acid secretion inhibitor, *iPTH* intact parathyroid hormone, *P* phosphate, *SD* standard deviation

^a^Analysis of covariance (covariate, baseline); model: change from baseline = [treatment] [type] [baseline] [treatment] * [type]

^b^Adjusted mean difference = [least square mean of FC] − [least square mean of placebo]

^c^*P* value: test for [adjusted mean difference of with GASI] vs [adjusted mean difference of without GASI]

The mean (standard deviation; SD) doses of FC in the safety analysis set were 3,240 mg/day (725) with GASI (*n* = 14) and 3,445 mg/day (831) without GASI (*n* = 46), respectively.

### Influence of GASI on patients under hemodialysis (GBA4-6)

**Fig. 5:**
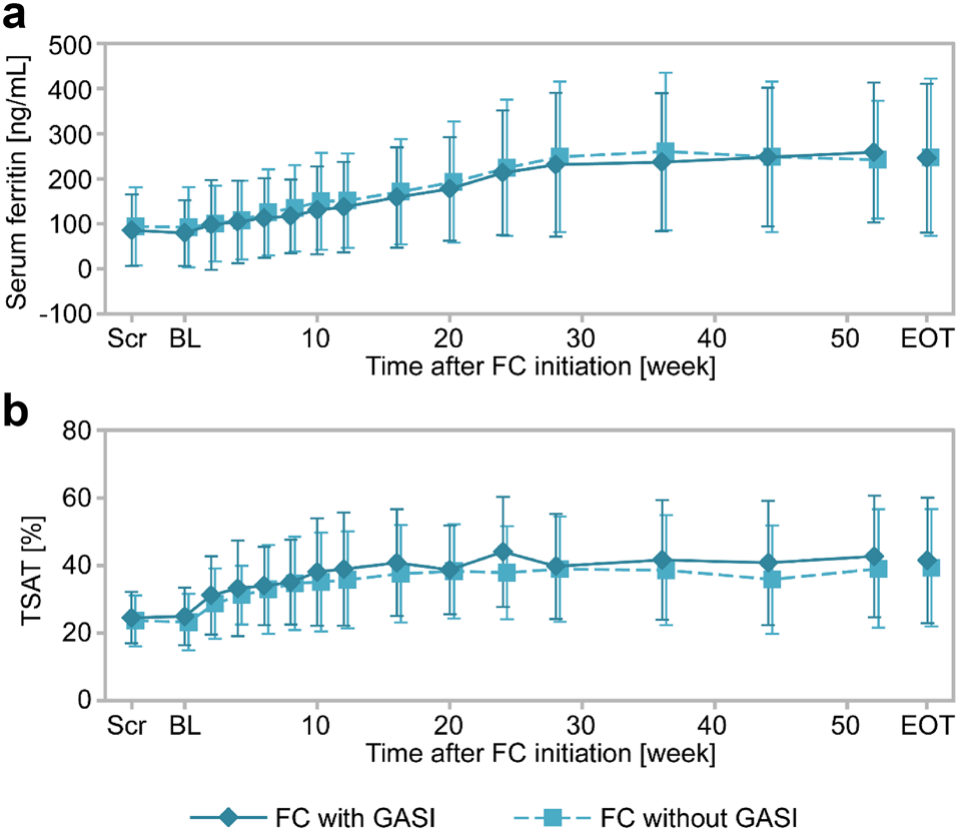
Time-course changes in serum ferritin and TSAT in patients undergoing hemodialysis from the GBA4-6 study (safety analysis set) Time-course changes in serum ferritin (**a**) and TSAT (**b**). Solid line, with GASI cohort; broken line, without GASI cohorts. Data are the mean ± standard deviation. *BL* baseline, *EOT* end of treatment, *FC* ferric citrate hydrate, *GASI* gastric acid secretion inhibitor, *Scr* screening, *TSAT* transferrin saturation

In GBA4-6, patients undergoing hemodialysis were treated with FC for 52 weeks. Time-course changes in iron-related parameters for with and without GASI cohorts are shown in Fig. 5. Serum ferritin (Fig. 5a) and TSAT (Fig. 5b) gradually increased during the treatment period and the levels were similar in with and without GASI cohorts. Changes from baseline to EOT in iron-related parameters and Hb are summarized in Table 3. Mean changes (SD) from baseline to EOT in serum ferritin were 166.32 ng/mL (153.70) in patients with GASI and 155.16 ng/mL (139.47) in patients without GASI, and those in TSAT were 16.60% (19.44) with GASI and 16.02% (18.81) without GASI. Changes in other parameters were similar in both cohorts.

**Table 3:** Changes in iron-related parameters and in mineral and bone disorder-related parameters in patients from the GBA4-6 study undergoing hemodialysis

Mean (SD)	With GASI, *n* = 95			Without GASI, *n* = 85		
	BL	EOT	Change	BL	EOT	Change
Serum iron [µg/dL]	61.5 (21.0)	85.5 (35.9)	24.0 (39.6)	58.5 (20.5)	84.2 (38.3)	25.6 (40.2)
Serum ferritin [ng/mL]	79.51 (73.10)	245.83 (165.33)	166.32 (153.70)	92.52 (88.95)	247.69 (174.60)	155.16 (139.47)
TIBC [µg/dL]	250.8 (44.6)	211.3 (34.6)	− 39.5 (35.0)	255.2 (43.6)	215.1 (32.2)	− 40.1 (31.3)
TSAT [%]	24.94 (8.50)	41.54 (18.57)	16.60 (19.44)	23.30 (8.38)	39.32 (17.34)	16.02 (18.81)
Hb [g/dL]	10.89 (1.00)	11.13 (1.26)	0.24 (1.43)	11.06 (1.07)	11.28 (1.26)	0.23 (1.18)
Serum P [mg/dL]	5.46 (1.13)	5.23 (1.07)	− 0.23 (1.41)	5.61 (1.36)	5.64 (1.53)	0.02 (1.69)
Serum cCa [mg/dL]	9.21 (0.59)	9.07 (0.63)	− 0.14 (0.56)	9.17 (0.55)	8.86 (0.58)	− 0.30 (0.57)
iPTH [pg/mL]	151.9 (107.0)	208.3 (130.8)	56.3 (119.0)	165.0 (144.6)	227.1 (144.5)	62.1 (129.0)
Ca*P [(mg/dL)^2^]	50.26 (10.66)	47.51 (10.43)	− 2.75 (13.07)	51.45 (12.72)	49.84 (13.42)	− 1.60 (14.84)

*BL* baseline, Ca*P calcium–phosphate product, *cCa* corrected calcium, *EOT* end of treatment, *GASI* gastric acid secretion inhibitor, *Hb* hemoglobin, *iPTH* intact parathyroid hormone, *P* phosphate, *SD* standard deviation, *TIBC* total iron-binding capacity, *TSAT* transferrin saturation

**Fig. 6:**
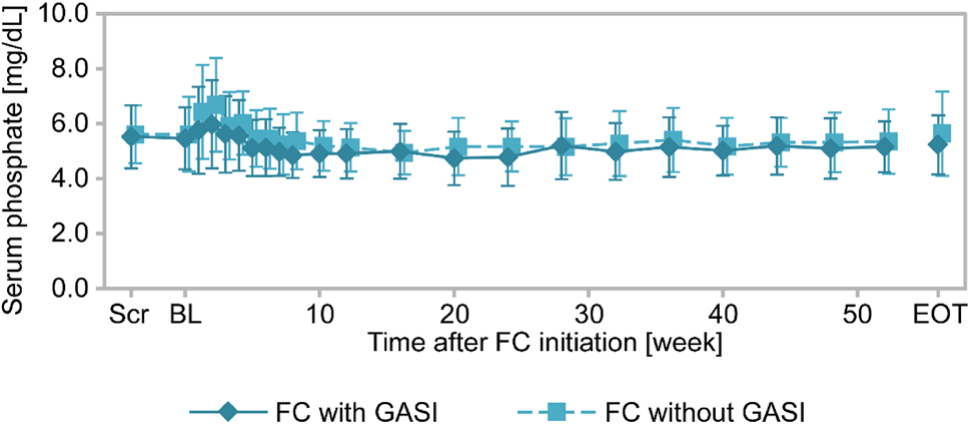
Time-course change in serum phosphate in patients undergoing hemodialysis from the GBA4-6 study (efficacy analysis set) Time-course changes in serum phosphate. Solid line, with GASI cohort; broken lines, without GASI cohorts. Data are the mean ± standard deviation. *BL* baseline, *EOT* end of treatment, *FC* ferric citrate hydrate, *GASI* gastric acid secretion inhibitor, *Scr* screening

Time-course changes in serum phosphate are shown in Fig. 6. The levels of serum phosphate were similar in with and without GASI cohorts throughout the observation period. The mean changes (SD) from baseline to EOT in serum phosphate were − 0.23 mg/mL (1.41) in patients with GASI and 0.02 mg/mL (1.69) in patients without GASI. Similarly, the changes from baseline to EOT in serum cCa, iPTH, and Ca*P were comparable between with and without GASI cohorts (Table 3).

The mean (SD) dose of FC in the safety analysis population was 2619 mg/day (1113) in the with GASI cohort (*n* = 95) and 2854 mg/day (1164) in the without GASI cohort (*n* = 85).

## Discussion

It is thought that the low pH of gastric acid is essential for the absorption of dietary iron and oral iron preparations, whether ferrous iron or ferric iron [1], and that the increase in intragastric pH by concomitant use of GASI might affect iron absorption from FC. Therefore, this study retrospectively investigated whether the iron absorption and phosphate-lowering effects of FC were influenced by GASI use. In the 12 week, randomized control study of non-dialysis-dependent CKD patients (GBA4-4), the FC group had a tendency towards increased serum ferritin and TSAT compared with placebo. This tendency was observed regardless of GASI use. Serum phosphate tended to be decreased in the FC group compared with placebo, regardless of GASI use. In the 52 week study of CKD patients undergoing hemodialysis (GBA4-6), the effect of FC to increase serum ferritin and TSAT was intact regardless of GASI use. Furthermore, FC maintained lower levels of serum phosphate regardless of GASI use. The dose of FC might strongly influence iron-related parameters, however, the mean doses of FC in both studies were similar between with or without GASI. This study demonstrated that GASI use considered not to influence on the iron absorption and phosphate-lowering effects of FC in patients with CKD.

The phosphate-lowering effect of other phosphate binders used to treat hyperphosphatemia in CKD patients undergoing hemodialysis, such as calcium carbonate and lanthanum carbonate, was hindered in patients with concomitant GASI use [16–18]. In the present study, we confirmed that the use of GASI did not interfere with the serum phosphate-lowering effect of FC in non-dialysis-dependent CKD patients and CKD patients undergoing hemodialysis, as predicted by a previous clinical study in patients with CKD undergoing hemodialysis treated with or without a concomitant histamine-2 receptor antagonist [13].

**Fig. 7:**
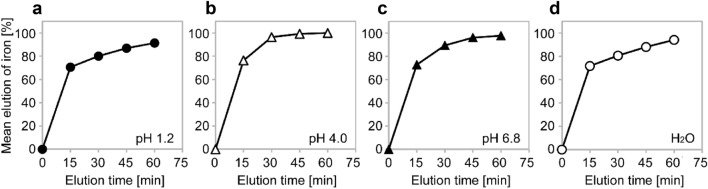
Elution behavior of iron from ferric citrate hydrate (Riona^®^) under different pH conditions. The elution solution was filtered through a 0.45 µm membrane filter, followed by reduction using L-ascorbic acid. The iron concentration was determined by the 1,10-phenanthroline spectrometric method and the elution rate was calculated using an iron standard solution (1000 mg/L Japan Calibration Service System), which was used as a 100% elution solution. The mean elution ratio of solutions adjusted for pH 1.2 (**a**), pH 4.0 (**b**), pH 6.8 (**c**), and a water control (**d**) (*n* = 6 for each). Modified from Koyama et al. [19]

A deficiency or overload of iron can cause serious health problems; therefore, to maintain homeostasis, iron absorption is strictly regulated. FC is a trivalent ferric iron, which must be reduced to divalent ferrous iron to be absorbed in the small intestine. The absorption of dietary ferric iron or iron preparations is reported to be lower than that of dietary ferrous iron or iron preparations because of their lower solubility and bioavailability [10, 20]. FC is formulated to have a large surface area for high solubility (32.4–39.9 m^2^/g for FC vs 0.62 m^2^/g for general ferric citrate products that are approved as a dietary supplement) [19], which may have resulted in the solubility at pH 6.8 being as rapid as that at pH 1.2 and the comparable elution behavior of iron from FC at pH 1.2, 4.0, and 6.8 (Fig. 7) [19]. Ferric Citrate (Auryxia^®^) which is a similar FC product and approved in the USA, the rate of dissolution at pH 8 was reported to be 3.08 times the rate of commercial-grade ferric citrate [11]. The properties of FC that allow it to be soluble at a high pH might enhance ferric iron absorption in the duodenum [10] and contribute to its effects on iron absorption and phosphate-lowering, as shown in this study.

FC achieves its iron absorption and phosphate-lowering effects via two contrasting mechanisms: ferric iron from FC, enzymatically reduced to ferrous iron, is absorbed in the small intestine [10, 20], and concurrently, ferric iron from FC binds to dietary phosphorus to form an insoluble complex that promotes the fecal excretion of phosphorus [5]. How this absorption–excretion balance is regulated is not known. Iron and phosphate are essential for the human body; therefore, they are thought to be regulated individually by such as hepcidin [21, 22] and fibroblast growth factor-23 [23, 24]. Gastrointestinal complications are known to occur in approximately 70% of patients with renal failure [25]. Accordingly, FC is considered to be effective in patients with CKD who are taking GASI not only as a phosphate binder but also as an iron preparation.

This was a retrospective study and the number of patients included was insufficient to make firm conclusions. Further prospective studies are needed and should include patients with iron deficiency anemia without CKD.

## Conclusions

This retrospective study using data from FC-treated CKD patients who were non-dialysis-dependent or were undergoing hemodialysis demonstrated that GASI use did not influence on the changes in iron-related parameters, such as serum ferritin and TSAT and serum phosphate by FC administration.

## Data Availability

The datasets generated and/or analyzed during the study are available from the corresponding author on reasonable request.
